# Adult Sox2+ stem cell exhaustion in mice results in cellular senescence and premature aging

**DOI:** 10.1111/acel.12834

**Published:** 2018-08-20

**Authors:** Jéssica M. Vilas, Carmen Carneiro, Sabela Da Silva‐Álvarez, Alba Ferreirós, Patricia González, María Gómez, Sagrario Ortega, Manuel Serrano, Tomás García‐Caballero, Miguel González‐Barcia, Anxo Vidal, Manuel Collado

**Affiliations:** ^1^ Laboratorio de Células Madre en Cáncer y Envejecimiento, Instituto de Investigación Sanitaria de Santiago de Compostela (IDIS) Xerencia de Xestión Integrada de Santiago (XXIS/SERGAS) Santiago de Compostela Spain; ^2^ Departamento de Fisioloxía and Centro de Investigación en Medicina Molecular (CIMUS), Instituto de Investigación Sanitaria de Santiago de Compostela (IDIS) Universidade de Santiago de Compostela Santiago de Compostela Spain; ^3^ Histopathology Core Unit Spanish National Cancer Research Centre (CNIO) Madrid Spain; ^4^ Trasgenic Mice Unit Spanish National Cancer Research Centre (CNIO) Madrid Spain; ^5^ Tumor Suppression Group Spanish National Cancer Research Centre (CNIO) Madrid Spain; ^6^ Institute for Research in Biomedicine (IRB Barcelona) The Barcelona Institute of Science and Technology (BIST) Barcelona Spain; ^7^ Catalan Institution for Research and Advanced Studies (ICREA) Barcelona Spain; ^8^ Departamento de Ciencias Morfológicas, Facultad de Medicina USC, Xerencia de Xestión Integrada de Santiago (XXIS/SERGAS) Santiago de Compostela Spain; ^9^ Servicio de Farmacia Xerencia de Xestión Integrada de Santiago (XXIS/SERGAS) Santiago de Compostela Spain

**Keywords:** Sox2, aging, adult stem cells, stem cell exhaustion

## Abstract

Aging is characterized by a gradual functional decline of tissues with age. Adult stem and progenitor cells are responsible for tissue maintenance, repair, and regeneration, but during aging, this population of cells is decreased or its activity is reduced, compromising tissue integrity and causing pathologies that increase vulnerability, and ultimately lead to death. The causes of stem cell exhaustion during aging are not clear, and whether a reduction in stem cell function is a cause or a consequence of aging remains unresolved. Here, we took advantage of a mouse model of induced adult Sox2+ stem cell depletion to address whether accelerated stem cell depletion can promote premature aging. After a short period of partial repetitive depletion of this adult stem cell population in mice, we observed increased kyphosis and hair graying, and reduced fat mass, all of them signs of premature aging. It is interesting that cellular senescence was identified in kidney after this partial repetitive Sox2+ cell depletion. To confirm these observations, we performed a prolonged protocol of partial repetitive depletion of Sox2+ cells, forcing regeneration from the remaining Sox2+ cells, thereby causing their exhaustion. Senescence specific staining and the analysis of the expression of genetic markers clearly corroborated that adult stem cell exhaustion can lead to cellular senescence induction and premature aging.

## 1 INTRODUCTION, RESULTS, DISCUSSION

Adult stem cell exhaustion is considered a hallmark of aging, and it is believed to be behind the progressive loss of physiological integrity that leads to the impaired tissue and organ function that results in the development of multiple pathologies collectively known as age‐related diseases (López‐Otín, Blasco, Partridge, Serrano, & Kroemer, 2013). It is not clear; however, if defective or reduced adult stem cell pools are responsible for the dysfunction of tissues in old organisms or, conversely, if aging causes a reduction in the number and/or functionality of the stem cells (Goodell & Rando, 2015; Schultz & Sinclair, 2016; Sharpless & DePinho, 2007).

SRY (sex determining region Y)‐box 2, Sox2, is a transcription factor of the Sox family with a crucial role regulating and maintaining selfrenewal and pluripotency in embryonic stem cells (Orkin et al., 2008; Rizzino, 2013). Apart from its role during early development, Sox2 is also expressed in progenitors at later stages of the mouse embryo (Graham et al., 2003; Doetzlhofer et al., 2006; Klassen et al., 2004; Aubert et al., 2003), and its expression persists in the adult organism in tissue stem cells of stratified and glandular epithelia of ectodermal and endodermal origin, as well as in sensory cells (Merkel and taste bud cells) and spermatogonial stem cells, where it has been proved to be a critical factor sustaining the homeostasis of these tissues (Arnold et al., 2011). Recently also, it has been shown that Sox2 expression diminishes with aging in several tissues in mice and humans (Carrasco‐Garcia et al., 2018). As Sox2 expression is critical for the normal regeneration and maintenance of numerous adult stem cell compartments, severe ablation of Sox2+ cells in adult mice results in the lethal disruption of tissue homeostasis. In an interesting manner, however, partial depletion of Sox2+ cells causes a reversible disruption of tissue integrity, and restoration of normal tissue homeostasis rescues morbidity. This recovery originates from the action of residual Sox2+ cells capable of reorganizing and regenerating the affected tissues (Arnold et al., 2011).

Here, we decided to test the stem cell exhaustion hypothesis of aging by subjecting transgenic mice to a protocol of partial repetitive depletion of adult Sox2+ cells. We reasoned that cycles of partial ablation and recovery could result in the exhaustion of the regenerating capacity of these Sox2+ cells and thus provided an excellent opportunity to test whether stem cell exhaustion results in premature aging.

**Figure 1:**
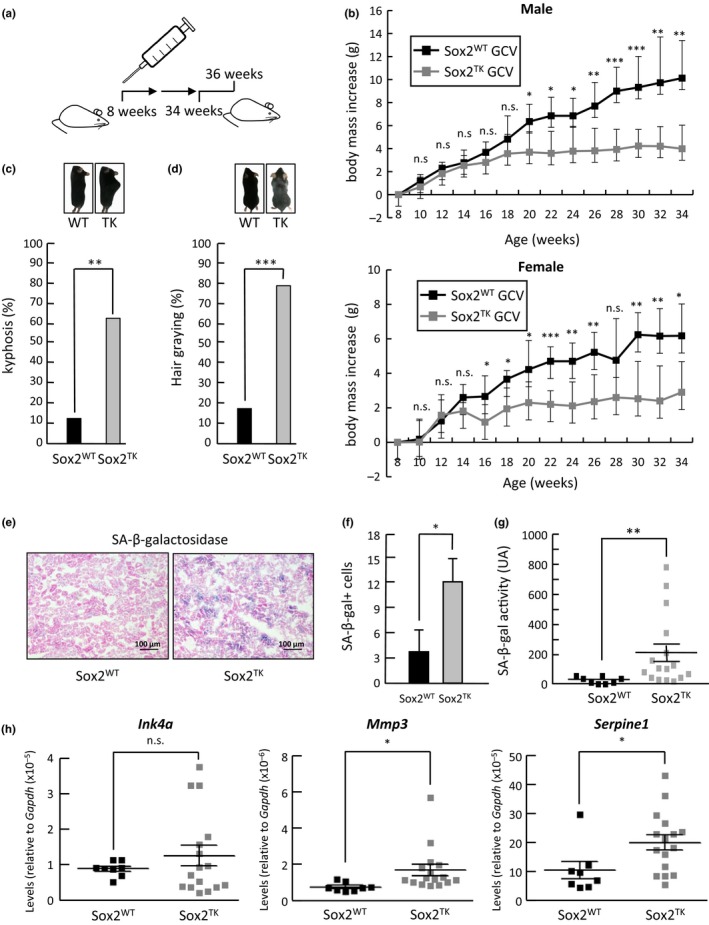
Short repetitive partial depletion of Sox2+ cells leads to defective growth and induction of senescence in mice. (a) Schematic representation of the repetitive partial depletion protocol in mice by intraperitoneal injection of GCV starting at 8 weeks, every 2 weeks, and until mice were 34 weeks. After treatment, mice were sacrificed at 36 weeks of age. (b) Body mass increase (g) in male (upper graph; *n* = 13) and female (lower graph; *n* = 11) control mice (Sox2WT; *n* = 8) or Sox2‐TK transgenic mice (Sox2TK; *n* = 16) treated with GCV along the experiment. (c) Quantification of animals (percentage, %) showing evident signs of kyphosis after GCV treatment. (d) Quantification of animals (percentage, %) showing evident signs of hair graying after GCV treatment. (e) SAbetaGal staining of kidney sections from control wild‐type (Sox2^WT^) and Sox2‐TK transgenic (Sox2^TK^) animals after GCV treatment. (f) Quantification of the number of SAbetaGal positive cells observed in stained kidney sections. (g) Chemiluminescence quantification of SAbetaGal activity using Galacton as a substrate. (h) Quantification of mRNA expression by QPCR of *Ink4a*,* Mmp3* and *Serpine1* in kidneys from control wild‐type (Sox2^WT^) and Sox2‐TK transgenic (Sox2^TK^) animals. Results are presented as mean ± *SD*. ***p < 0.001, **p < 0.01,*p < 0.05, n.s. nonsignificant

We took advantage of transgenic Sox2‐TK mice, in which herpes simplex virus type‐1 (HSV1) thymidine kinase (TK) gene was inserted into the *Sox2* allele (Arnold et al., 2011). A similar mouse model previously generated in the laboratory of Konrad Hochedlinger was successfully used to prove that adult Sox2+ cells are stem and progenitor cells in a number of tissues (Arnold et al., 2011). Once we obtained these mice, we decided to confront Sox2‐WT mice versus Sox2‐TK mice, both treated with GCV. Starting at 8 weeks of age, mice received intraperitoneal injections of GCV every 2 weeks and until they were 34 weeks old, for a total of 14 injections (Figure 1a). We followed the increase in body mass along the experiment and observed that Sox2‐TK mice treated with GCV were not increasing their body mass as much as GCV‐treated WT animals, and this was happening for both, male and female mice (Figure 1b). When the treatment was stopped, we measured body mass composition using echoMRI and found that Sox2‐TK mice treated with GCV had a reduced fat mass compared to Sox2‐WT mice, although this difference only reached statistical significance for male mice (Figure S1). We also confirmed the reduction in the number of Sox2+ cells, and the effects produced by the partial depletion in tissues previously described as dependent on the activity of stem and progenitor Sox2+ cells. We inspected the testis (Arnold et al., 2011), esophagus and trachea (Zhang et al., 2017) and found that GCV‐treated mice were showing atrophic seminiferous tubules (Figure S2), a feature previously described for these transgenic mice after acute depletion of Sox2+ cells (Arnold et al., 2011) and a frequent event during aging (Gosden, Richardson, Brown, & Davidson, 1982), and a decreased epithelial cellularity in esophagus and trachea that corresponded with a reduced number of Sox2+ cells (Figure S2).

During GCV treatment, visual inspection of mice revealed obvious signs of aging such as pronounced spinal kyphosis and hair graying (Harkema, Youssef, & Bruin, 2016). We evaluated these parameters at the end of the treatment and found a significantly higher proportion of animals showing kyphosis in the Sox2‐TK treated group compared to the Sox2‐WT group (Figure 1c). Also, the percentage of animals showing gray hair was significantly higher in the Sox2‐TK group treated with GCV compared to the Sox2‐WT treated mice (Figure 1d). At last, we decided to test hair regrowth capacity of these animals as a surrogate functional readout of aging of the animals (Finch, 1973). Sox2‐TK mice treated with GCV showed a clear reduction in their potential to regrow hair after plucking, with a majority of animals completely unable of regrowing hair after 15 days, compared to Sox2‐WT treated mice (Figure S3), pointing to a functional defect in Sox2‐TK mice after treatment.

Since the gradual accumulation of senescent cells in tissues with age is a hallmark of aging (Jeyapalan & Sedivy, 2008), we decided to assess the number of cells undergoing senescence after partial repetitive Sox2+ cell exhaustion in the Sox2‐TK and Sox2‐WT mice treated with GCV. For this, we analyzed the expression of the most widely used marker of the process, the senescence‐associated beta‐galactosidase activity (SAbetaGal) (Dimri et al., 1995) in kidneys from treated animals, an organ typically showing a clear age‐related deterioration. When we used frozen kidney sections to perform SAbetaGal staining reactions, we observed a clear positive X‐Gal staining indicative of induction of cell senescence that appeared higher in GCV‐treated Sox2‐TK mice compared to Sox2‐WT mice (Figure 1e). It is interesting that Sox2 is not expressed in the kidney suggesting that a systemic response is triggered by the exhaustion of the Sox2+ cells, causing the induction of cell senescence at distant tissues. Quantifications of senescent positive cells corroborated this observation (Figure 1f). To confirm this result, we used an alternative substrate that produces a luminescent reaction, Galacton (Bassaneze, Miyakawa, & Krieger, 2008), instead of a color reaction obtaining very similar results (Figure 1g). In addition, we measured the expression levels of several senescence marker genes: cell cycle inhibitor *Ink4a* (Krishnamurthy et al., 2004), matrix metalloprotease *Mmp3* (Komosinska‐Vassev et al., 2011), and the serine protease inhibitor *Serpine1* (Goldstein, Moerman, Fujii, & Sobel, 1994; Mu & Higgins, 1995). The mRNA levels of these genes were elevated in Sox2‐TK mice after GCV treatment, although in the case of *Ink4a* the increase did not reach statistical significance (Figure 1h), corroborating the induction of senescence in Sox2‐TK tissues.

**Figure 2:**
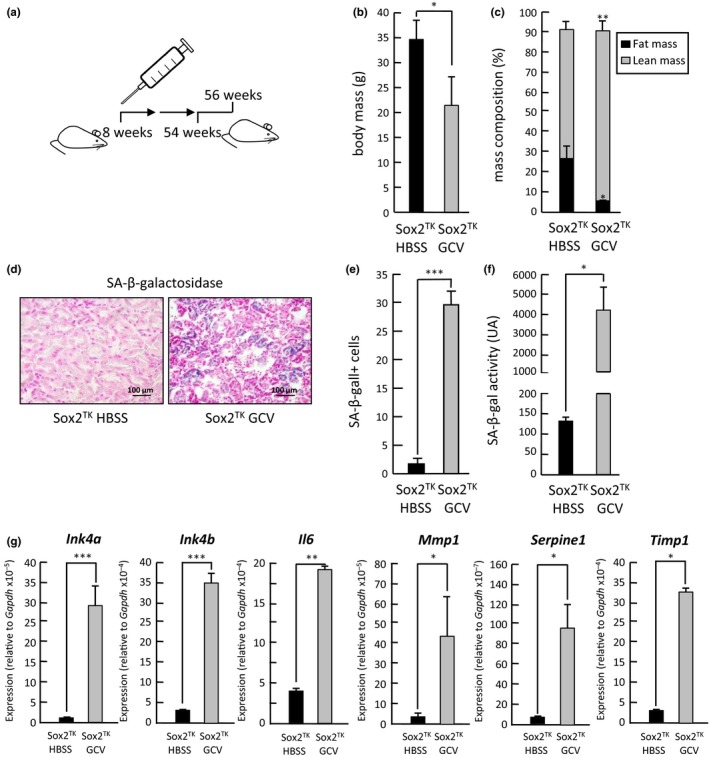
Prolonged repetitive partial depletion of Sox2+ cells leads to induction of senescence and premature mice. (a) Schematic representation of the prolonged repetitive partial depletion protocol in mice by intraperitoneal injection of GCV starting at 8 weeks, every 2 weeks, and until mice were 54 weeks. After treatment, mice were sacrificed at 56 weeks of age. (b) Quantification of body mass (g) in Sox2‐TK transgenic animals after GCV (*n* = 5) or vehicle (HBSS; *n* = 2) treatment. (c) Quantification of relative body mass composition (% of fat and lean mass) in Sox2‐TK transgenic animals after GCV or vehicle (HBSS) treatment. (d) SAbetaGal staining of kidney sections from Sox2‐TK transgenic animals after GCV or vehicle (HBSS) treatment. (e) Quantification of the number of SAbetaGal positive cells observed in d. (f) Chemiluminescence quantification of SAbetaGal activity using Galacton as a substrate. (g) Quantification of mRNA expression by QPCR of *Ink4a*,* Ink4b*,* Il6*,* Mmp1*,* Serpine1,* and *Timp1* in kidneys from Sox2‐TK transgenic animals after GCV or vehicle (HBSS) treatment. Results are presented as mean ± *SD*. ***p < 0.001, **p < 0.01,*p < 0.05, n.s.: nonsignificant

We reasoned that the premature aging phenotype that we were observing was only partial and moderate probably due to the short protocol of GCV treatment under which we were examining the effect of the partial depletion of adult progenitor/stem Sox2+ cells. To test this hypothesis, we decided to subject our mice to a longer protocol of around 1 year of repetitive partial depletion of Sox2+ adult cells by injecting GCV intraperitoneally in Sox2‐TK mice every 2 weeks, starting at 8 weeks of age and until they were 54 weeks old (Figure 2a). Control mice were equally injected with vehicle, HBSS. During the course of the experiment, we observed a progressive decline in spontaneous activity and exploratory behavior in the GCV‐treated mice. We stopped GCV administration after 24 injections and sacrificed the animals when they were 56 weeks of age. GCV‐treated mice showed a clearly reduced total body mass (Figure 2b), and when we determined lean to fat body mass using echoMRI, we observed a clear reduction of fat mass (Figure 2c) in GCV‐treated mice compared to HBSS control mice, in agreement with our observations for the shorter depletion protocol. We then analyzed the expression of SAbetaGal to test for the accumulation of senescent cells in kidney tissue sections and observed a strong positive staining in samples from GCV‐treated mice compared to control samples that were, for the most part, negative (Figure 2d). Indeed, quantification of SAbetaGal positive cells in various sections from kidneys extracted from different animals revealed a massive induction of cell senescence after GCV treatment (Figure 2e). To confirm this result, we used again the alternative luminescence substrate for SAbetaGal activity, Galacton. In agreement with the X‐Gal data, Galacton revealed a massive induction of senescence in the kidneys of GCV‐treated mice (Figure 2f). We also measured the mRNA expression of different genes linked to the senescent cell response, such as *Ink4a* (Krishnamurthy et al., 2004), *Ink4b* (Malumbres et al., 2000), *Il6* (Acosta et al., 2008; Kuilman et al., 2008), *Mmp1* (Benanti, Williams, Robinson, Ozer, & Galloway, 2002), *Serpine1* (Goldstein et al., 1994; Mu & Higgins, 1995), and *Timp1* (Komosinska‐Vassev et al., 2011). In all cases, we observed a statistically significant increase in the levels of mRNA for these genes, in agreement with the notion of a strong induction of senescence, a response typically associated with advanced aging (Figure 2g).

These observations demonstrate that promoting adult stem cell depletion can lead to a systemic response that can trigger senescence induction and premature aging of tissues, pointing to stem cell exhaustion as a causal factor in physiological aging.

## CONFLICT OF INTEREST

None declared.
