# Supplementary material for: Adult Sox2+ stem cell exhaustion in mice results in cellular senescence and premature aging
Source: Aging Cell. 2018 Aug 20;17(5):e12834. doi: 10.1111/acel.12834 (PMC6156495; doi:10.1111/acel.12834)
Supplement: Supplementary file 1 [file ACEL-17-e12834-s001.docx]

**SUPPLEMENTAL INFORMATION**

**MATERIALS AND METHODS**

**Sox2-TK mice and treatments**

V6.5 ESCs targeted with knockin constructs containing a puro-delta-TK (Chen and Bradley, 2000) allele under the control of endogenous Sox2 (previously described in Arnold et al., 2011) were kindly provided by Dr Konrad Hochedlinger. We functionally tested the HSV-TK expressing ES cells by culturing them for 6 days in the presence of ganciclovir (0.25 μg/mL). After this period of time almost all the Sox2-TK ESCs were dead while the control ESCs were unaffected.


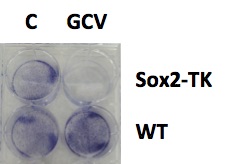


Correct clones were injected into C57BL/6 blastocysts and transferred into pseudopregnant females. Resultant chimeric mice were bred with C57BL/6 mice and germline offspring were bred to establish stable lines. These genotypes were maintained on a C57BL/6 and 129SvJ hybrid background and all comparisons were made among mice derived from the same sets of crosses, and they therefore shared the same genetic background.

Starting at 8 weeks of age, Sox2-TK mice were intraperitoneally injected with ganciclovir (GCV) at 100 mg/Kg in HBSS (Cymevene, Roche Pharmaceuticals) or vehicle (HBSS) every 2 weeks during the first 34 or 54 weeks of life, to complete a total of 14 or 24 injections, respectively.

Animals were kept under SPF conditions and all experiments were approved by the Santiago de Compostela University Bioethics Committee (protocol number 15005AE/07/01/02/05C/AVF2) in compliance with Principles of Laboratory Animal Care of national laws.

**Evaluation of body mass**

The body mass of males and females of 36 weeks-old mice was measured digitally every two weeks from the beginning (8 weeks) until the end of the treatment (34 weeks), being finally represented as body mass increase (g). In the case of the 56 weeks-old mice, total body mass (g) was digitally registered at 54 weeks.

For the evaluation of the body composition (fat and lean mass), Sox2-TK mice were individually scanned using MRI (EchoMRI^TM^-100H, *Whole Body Composition Analyser*, Echo Medical System). Percentages of fat and lean mass were calculated as fat or lean mass (g)/body weight (g) x 100. MRI scanning was performed at 34 or 54 weeks of age, for the short or long depletion protocols, respectively.

**Evaluation of kyphosis and hair graying**

The degree of kyphosis and hair graying was determined from digital photographs and a semiquantitative assessment using a scale from 0 to 1 (one being the presence of the feature) in Sox2WT (n=18) and Sox2TK (n=18) mice. The scoring was done blindly by three investigators independently who obtained essentially identical scores.

**Hair re-growth assay**

To perform the hair re-growth assay, dorsal hair was removed by plucking from a square of approximately 1.5 cm × 1.5 cm in Sox2WT (n=5) and Sox2TK (n=12). Hair re-growth was determined 15 days later based on digital photographs and a semiquantitative assessment using a scale from 1 to 3 (three being complete hair regeneration). Scoring was done blindly by three investigators independently who obtained essentially identical scores.

**Quantitative RT-PCR**

To measure RNA expression, total RNA was extracted using the NucleoSpin® RNA kit (Macherey- Nagel) following the indications of the provider and DNAse treatment. After nanodrop RNA quantification, the RNA was retrotranscribed into cDNA according to the manufacturer’s protocol (High-Capacity cDNA Reverse Transcription Kit, Applied Biosystems). Quantitative Real Time-PCR was performed using SYBR Green Power PCR Master Mix (Applied Biosystems) in an AriaMx real-time PCR system (Agilent technologies). Relative quantitative RNA was normalized using the housekeeping gene *Gapdh.* The primers used for Quantitative Real Time-PCR were:

*Ink4a*-F: 5’-CGTACCCCGATTCAGGTGAT-3’

*Ink4a*-R: 5’-TTGAGCAGAAGAGCTGCTACGT-3’

*Ink4b*-F: 5’-AGATCCCAACGCCCTGAAC-3’

*Ink4b*-R: 5’-CCCATCATCATGACCTGGATT-3’

*Il6*-F: 5’-GCTACCAAACTGGATATAATCAGGA-3’

*Il6*-R: 5’-CCAGGTAGCTATGGTACTCCAGAA-3’

*Mmp1*-F: 5’-CCTCGTTGGACCAAAACACA-3’

*Mmp1*-R: 5’-GCGATGGCATCTTCCACAA-3’

*Mmp3-F*: 5’-CAAAACATATTTCTTTGTAGAGGACAA-3’

*Mmp3-R*: 5’-TTCAGCTATTTGCTTGGGAAA-3’

*Serpine1*-F: 5’-TTGAATCCCATAGCTGCTT-3’

*Serpine1*-R: 5’-GACACGCCATAGGGAGAGA-3’

*Timp1*-F: 5’-CGGAAATTTGCACATCAGTG-3’

*Timp1*-R: 5’-GACCTGATCCGTCCACAAAC-3’

**Tissue preparation and histological analysis**

Sox2-TK mice were sacrificed when they were 36 or 56 weeks old and tissues were removed. For Senescence-Associated beta-Galactosidase (SAbetaGal) staining, kidneys were rapidly frozen in liquid nitrogen and mounted in OCT. Sections (10 μm) were cut and mounted onto glass slides to perform later SAbetaGal staining. Counterstaining was performed with Nuclear Fast Red (NFR) staining and slides were evaluated on a Zeiss Axiovert microscope under bright field at 20X magnification. For immunohistochemical analysis, tissues were fixed in formalin at 4ºC, embedded in paraffin wax, and sectioned at a thickness of 5 μm. Sections were stained with hematoxylin and eosin for pathological examination or processed for immunohistochemical analysis with an antibody against mouse Sox2 (CST #3728, C70B1) and viewed under bright field at 20-40X magnification. In both cases, the number of positive cells was quantified using ImageJ software (NIH).

**Senescence-Associated beta-Galactosidase (SA-betaGal) activity assays**

A colorimetric and a chemiluminescent staining assay were performed to detect SAbetaGal activity. For the colorimetric staining, kidney sections were washed in PBS, fixed for 25 min (room temperature) in 2% formaldehyde/0.2% glutaraldehyde, washed, and incubated overnight at 37°C with fresh SAbetaGal staining solution: 1 mg of 5-bromo-4-chloro-3-indolyl beta-D-galactoside (X-Gal) per mL (Fisher Scientific), 40 mM citric acid/sodium phosphate pH 5.5, 5 mM K_3_Fe[CN]_6_ , 5 mM K_4_Fe[CN]_6_, 150 mM NaCl, and 2 mM MgCl_2_ (Dimri et al., 1995). For chemiluminescent detection, 30 mg kidney protein extracts were used to perform this assay with the Galacto-Light Plus™ beta-Galactosidase Reporter Gene Assay System Kit (Applied Biosystems) according to the manufacturer’s instructions but using a citric acid/sodium phosphate buffer at pH 5.5. The luminescence of the samples was measured using the Mithras LB 940 multimode microplate reader (Berthold Technologies).

**Statistical analyses**

The statistical significance of the data obtained was analyzed using the two-tailed *Student's* *t*-test or the *Fisher's* exact test: *** *p* < 0.001; ** *p* < 0.01; * *p* < 0.05; n.s. not significant, except for kyphosis, hair graying and hair re-growth for which we used two-tailed Chi-square.

**REFERENCES FOR METHODS**

Arnold, K., Sarkar, A., Yram, M.A., Polo, J.M., Bronson, R., Sengupta, S., Seandel, M., Geijsen, N., and Hochedlinger, K. (2011). Sox2+ Adult Stem and Progenitor Cells Are Important for Tissue Regeneration and Survival of Mice. Cell Stem Cell *9*, 317–329.

Chen, Y.T., and Bradley, A. (2000). A new positive/negative selectable marker, puDeltatk, for use in embryonic stem cells. Genesis *28*, 31–35.

Dimri, G.P., Lee, X., Basile, G., Acosta, M., Scott, G., Roskelley, C., Medrano, E.E., Linskens, M., Rubelj, I., and Pereira-Smith, O. (1995). A biomarker that identifies senescent human cells in culture and in aging skin in vivo. Proc. Natl. Acad. Sci. U. S. A. *92*, 9363–9367.


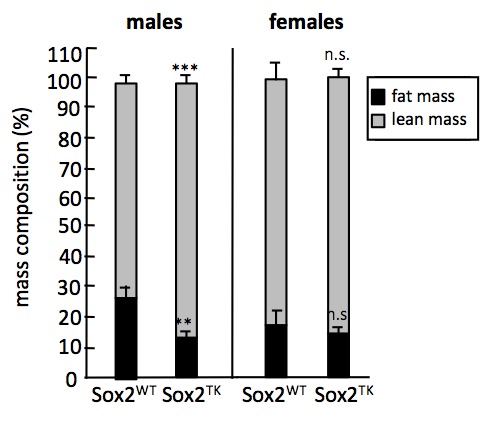


**Supplemental figure S1.**  Relative body mass composition (%) analysis after completion of GCV treatment at 36 weeks of age showing fat and lean mass in male and female control (Sox2^WT^) or transgenic (Sox2^TK^). Animals received intraperitoneal injection of GCV starting at 8 weeks, every 2 weeks, and until mice were 34 weeks.


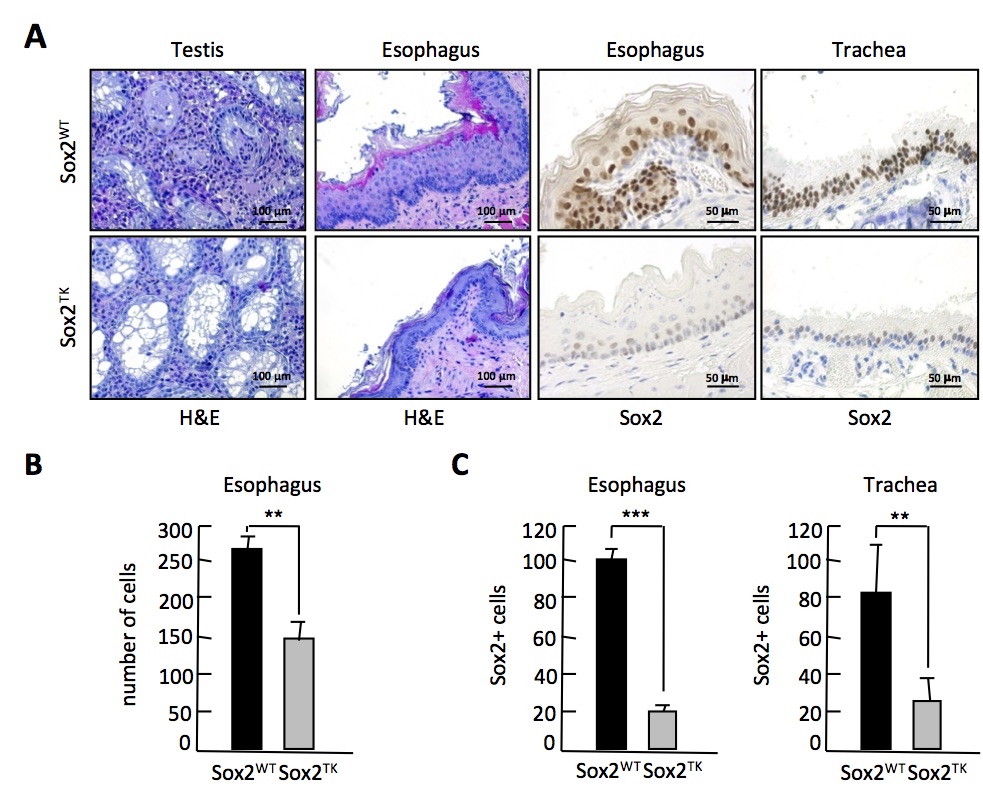


**Supplemental figure S2.** (**A**) Histological analysis of H&E stained sections from testis and esophagus, and immunohistochemistry for Sox2 on sections from esophagus and trachea, from GCV-treated control wild type (Sox2^WT^, upper panels) or Sox2-TK (Sox2^TK^, bottom panels) mice. (**B**) Quantification of number of cells in the epithelial layer of the esophagus of control wild type (Sox2^WT^) or Sox2-TK transgenic (Sox2^TK^) animals treated with GCV. (**C**) Quantification of Sox2+ cells in the epithelial layer of the esophagus (left panel) and trachea (right panel) of control wild type (Sox2^WT^) or Sox2-TK transgenic (Sox2^TK^) animals treated with GCV.


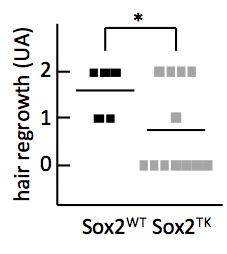


**Supplemental figure S3.**  Evaluation of the capacity to re-grow hair after plucking in control wild type (Sox2^WT^) and Sox2-TK transgenic (Sox2^TK^) animals after GCV treatment.
